# Biofabrication of Bacterial Constructs: New Three-Dimensional Biomaterials

**DOI:** 10.3390/bioengineering6020044

**Published:** 2019-05-14

**Authors:** Amin Shavandi, Esmat Jalalvandi

**Affiliations:** 1 BioMatter-Biomass transformation Lab (BTL), École interfacultaire de Bioingénieurs (EIB), Université Libre de Bruxelles, Avenue F.D. Roosevelt, 50 - CP 165/61, 1050 Brussels, Belgium; 2 School of Engineering and Physical Sciences, Heriot-Watt University, Edinburgh EH14 4AS, UK; e.jalalvandi@hw.ac.uk

**Keywords:** bacteria biofabrication, 3D printing, tissue engineering, probiotic food

## Abstract

An enormous number of bacteria live in almost every environment; from deep oceans to below the surface of the earth or in our gastrointestinal tract. Although biofabrication is growing and maturing very quickly, the involvement of bacteria in this process has not been developed at a similar pace. From the development of a new generation of biomaterials to green bioremediation for the removal of hazardous environmental pollutants or to develop innovative food products in a recent trend, researchers have used cutting-edge biofabrication techniques to reveal the great potential of 3D structured bacterial constructs. These 3D bacterial workhouses may fundamentally change our approach toward biomaterials.

We are a giant symbiont organism composed of Homo sapiens and microbial cells, while the microbes in our body have a larger genome size than us [1,2]. We are learning more and more about the significance of bacteria in our body and their communication with organs such as liver and brain. An incredible recent study found a key role of bacteria in the development of schizophrenia [3]. Biofabrication in recent years progressed toward the concept of an automated development of structured materials with biological function. Living cells, bioactive molecules and hybrid cell-material structures have developed through biofabrication, among others [4]. However, bacteria traditionally have not been largely considered in biofabrication, and their enormous potential for the development of functional 3D biomaterials has mainly remained unknown. In an early study, Weible and co-workers [5] printed patterns of bacteria on the agar surface in a petri dish using soft lithography. For this purpose, agarose stamps were fabricated by casting agarose solutions on the Polydimethylsiloxane (PDMS) moulds. By applying cell suspension, the cells were deposited on the surface of the stamp which was then used for transferring the pattern of bacteria to agar plates containing culture media. The authors could use one single stamp to print a bacterial pattern at least 250 times on agar culture. The reported agarose stamp technique is capable of patterning various strains of bacteria in a simultaneous, reproducible and rapid process. The proposed stamping technique can be useful for different scientists interested in developing a pattern of bacteria on cell culture media in order to study the organisms’ interaction with each other, molecules or the material surface (Figure 1A).

To better understand the cell–cell interactions in a complex microbial environment besides gaining insight into the role of geometry on the bacterial pathogenicity, Connell et al. [6] proposed a new 3D printed cellular model using a laser-based lithography method. Applying this technique, selected bacteria were trapped and sealed within the cavities formed by the crosslinked chains of gelatin. The authors showed the interaction between human pathogens of *Staphylococcus aureus* and *Pseudomonas aeruginosa* in a 3D structure indicating the survival of *S. aureus* from antibiotic treatment with β-lactam when enclosed in 3D shell communities composed of *P. aeruginosa*. Given that a bacterial community thrives in a 3D structure in the human body, the proposed technique can be useful to study the role of geometry in pathogenicity.

The incorporation of desired proteins such as bovine serum albumin (BSA) to the gel can enhance the mechanical and chemical properties of the 3D structure, and it is possible to print various cell types using different fabrication gels (Figure 1B). The ability of bacteria to generate new materials was combined with the tunable properties of the 3D printing method by Lehner and co-workers [7], who fabricated the 3D structure of bacterial cultures leading to the development of new sustainable materials. Using a modified commercial 3D printer, a mixture of bacteria and alginate was extruded, cross-linked and formed a gel upon contact with a calcium ion containing surface resulting in the preparation of 3D microbial structures in a reproducible manner. A high printing resolution was achieved using this technique, and the rate of extrusion and print head speed were reported as two major parameters affecting the printing resolution. The developed system allowed printing multilayer bacterial structures. Two different strains of *Escherichia coli* able to express proteins at distinct colours were printed in a bilayer structure (Figure 2A). The analysis indicated a good separation in addition to bacterial survival and metabolic activity in the gel layers up to 48 hours of incubation. The proposed printing system can be used for the preparation of different bacteria containing materials in a patterned format within millimetre resolution. Nevertheless, limitations of the system include the production of 3D printed structures with internal bridges or hollow spaces. In addition, it is not yet possible to directly process bacteria in a complex 3D structure. The development of biofilm is also not controlled, and the chemistry of the matrix polymer can be the limiting factor regarding the stability of the developed bacterial structure.

By combining genetic engineering and 3D printing, the same group in a recent study [8] developed a standardised and reproducible method for the production of 3D biofilm structures. A low-cost 3D printer “The Biolinker” was utilised to print bacterial suspension in an alginate solution which turns into a gel on a substrate containing calcium. The authors printed engineered *E. coli* that in the presence of an inducer produces biofilm and the biofilm formation could be controlled by the genetic control of a gene (csgA). These 3D printed biofilms could have diverse functions and applications, such as sequestration of metal ions or water filtration. Schaffner and co-workers [9] proposed a 3D printing system to develop cell-laden hydrogels called “Flink” with the ability to control the cells’ concentration and their spatial distribution in the 3D structure. The developed biocompatible hydrogel synthesised from nontoxic substances of k-carrageenan, hyaluronic acid and fumed silica had the viscoelastic properties suitable for the immobilisation of the cells and the production of 3D printed structures through multilateral direct ink writing (DIW). The authors showed the capability of the designed system with two examples of 3D structures for bioremediation and biomedical applications. In the first example, in order to benefit from phenol degrading capability of *Pseudomonas putida*, this bacterium was immobilised and 3D-printed. The 3D bacterial lattice structure with the high surface area could degrade the phenol as a major and toxic substance without the need for a supporting material. The degradation of phenol was found to be caused by bacteria that have been released from the 3D structure in the phenol-containing medium as well as the cells immobilised on the surface of the 3D structure. In the second example, Flink was loaded with *acetobacter xylinum* for the in situ production of cellulose in the form of a 3D structure with good mechanical properties, making it suitable for biomedical applications. In this case, once the bacteria have produced cellulose, the ink residue was washed away, leaving a cellulose network with a specific geometry and topography (Figure 2B).

In line with the potential of 3D printed bacteria for bioremediation applications, Qian et al. [10] developed living inks using freeze-dried baker’s yeast and printed catalytically active structures at a high resolution (100 µm) through direct ink writing techniques. The cell-containing ink had shear-thinning rheological behaviour suitable for extrusion 3D printing. Printed cell structures can ferment glucose and produce ethanol and CO_2_. The proposed ink system in this study can be used for printing different microbes with catalytic activities for a broad range of biotechnological applications. The food industry can also benefit from the 3D fabrication of bacteria. In a different direction, in a recent study, Zhang and colleagues [11] investigated the possibility of manufacturing 3D printed cereal-based food, loaded with probiotic bacteria *Lactobacillus plantarum* WCFS1. Like hydrogel composition—which affects viscosity and printability—the dough formulation, the flour type and water content determine the feasibility of printing the dough in 3D structures. The probiotic loaded bacterial structures were printed using a fused deposition modelling method in two honeycomb and concentric structures (Figure 3) and baked at different temperature of 145, 175 and 205 °C. One major obstacle to produce bakery products containing probiotic bacteria is the survival of the organism at the baking temperature. In this regard, increasing the rate of drying of the products can help shorten the baking time. Therefore, food structures with high surface-to-volume ratios produced by the 3D printing approach can be beneficial for this purpose. The authors have incorporated sodium caseinate to increase the viscoelastic properties of the dough to enhance its printability. The probiotics could survive the baking process of 6 min at 145 °C in honeycomb structures, and by having more than 106 CFU/g, the baked product could be defined as a probiotic food. The results reported in this study may offer a new avenue to the development of innovative bakery products containing probiotics.

Microbial biofilm models are the major form of microbial life, which are composed of a microorganism and extra polysaccharides. These 3D structures can be used to evaluate microbial metabolism and their interaction with the surrounding media. Recent research at Montana State University focused on engineering 3D printed biofilms of methanotroph bacteria with specific characteristics in order to convert methane into various organic materials, such as bioplastics. This can result in the reduction of the methane emitted into the environment and at the same time result in the development of bio-based materials as an intersection of technology and nature [10]. In another study, a microporous 3D bacterial cellulose foam was developed through foaming, and bacterial cellulose was formed directly at the air–water interfaces of the air bubble (Figure 4A) [11]. The authors used Cremodan as a surfactant for foaming and Xanthan as a thickener to obtain the stable foam. The biocompatible 3D structured bacterial cellulose may have potential applications in skin tissue engineering and wound healing which addresses the lack of porosity of the bacterial cellulose conventionally produced.

In a recent creative study [12], Manoor’s lab 3D printed colonies of cyanobacteria and graphene nanoribbons on biotic and abiotic (polysiloxane) mushroom pileus and created bionic mushrooms (Figure 4B). The highly packed 3D printed bacteria could nourish from the mushroom for generating photosynthetic electricity which was collected using graphene nanoribbons. Interestingly, the biotic mushroom provided bacteria a suitable environment (e.g., temperature, pH and moisture) helping toward their viability, while these conditions were absent in abiotic mushroom resulting in less viability of the bacteria.

The work highlighted the possibility to harness the benefit of 3D printed bacteria to realise an environmentally friendly source of photosynthetic electricity [12].

In conclusion, in this perspective, we have summarised the state-of-the-art biofabrication of bacterial constructs, highlighting the progress and unmet challenges. The potential application of 3D printed bacterial constructs is diverse, ranging from studying the development of infection in vivo to producing 3D structured probiotic foods, converting methane into bioplastics, producing photosynthetic electricity and biomedical applications. Despite these intriguing studies and reports, the current 3D bioprinters are slow and operate at small scales. Future studies are required to develop new 3D bioprinters which are affordable, scalable and able to print different types of bacterial inks with diverse viscosities at a short time and in a controlled fashion. Considering the high and growing demand for green products and the potential applications of 3D printed bacterial constructs, it is highly predictable that those barriers will soon be resolved.

## Figures and Tables

**Figure 1:**
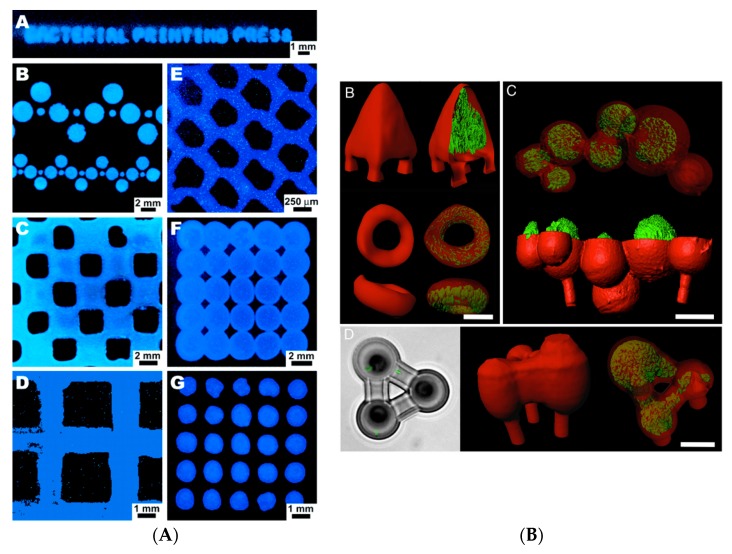
(**A**) Various patterns of *Vibrio fischeri* colonies printed on the agar surface in a petri dish using soft lithography. Adapted with permission from [5]. Copyright (2005) American Chemical Society. (**B**) Confocal fluorescence images show gelatin-based micro-3D printing in the presence of Pseudomonas aeruginosa microcolonies [6].

**Figure 2:**
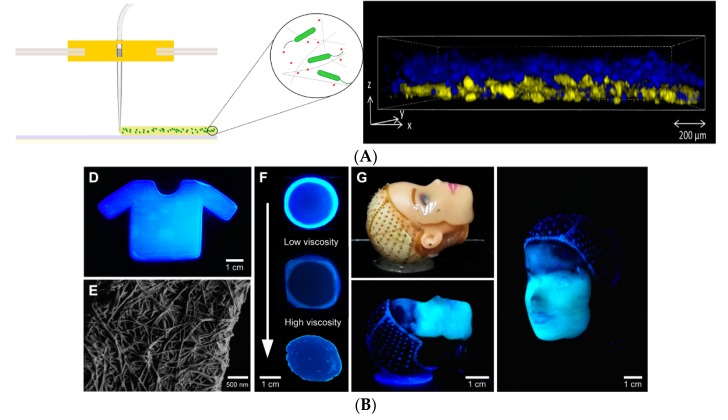
(**A**) Modified strains of *Escherichia coli* contain blue fluorescent and yellow cells printed in a layered structure using a modified commercial 3D printer. (ACS Author Choice—This is an open access article published under a Creative Commons Non-Commercial No Derivative Works (CC-BY-NC-ND) Attribution License). (**B**) 3D-printed complex structures containing bacteria for various bioremediation and biomedical applications [8]; Open-access article distributed under the terms of the Creative Commons Attribution-Non Commercial license.

**Figure 3:**
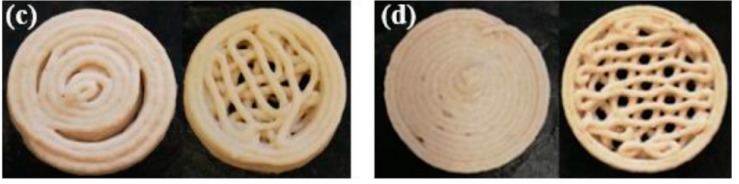
3D printing dough formulations with different nozzle sizes of 1.6 mm (**left**) and 1.2 mm (**right**) containing probiotic bacteria [9].

**Figure 4:**
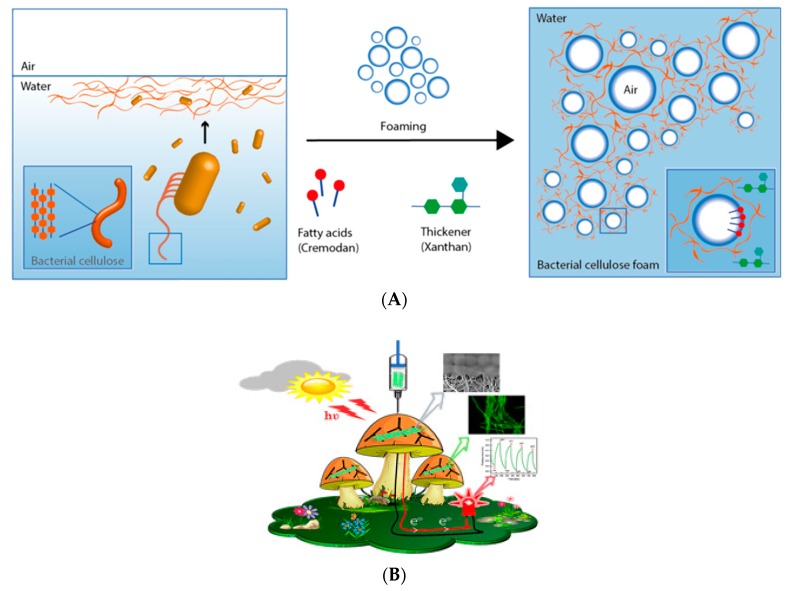
(**A**) Schematic of the formation of bacterial cellulose foam through foaming of a mannitol-based media with a bacterial suspension of *Gluconoacetobacter xylinus.* Cremodan as a surfactant stabilises the foam. Xanthan provides stability to the foam [11] (Open access). (**B**) Schematic drawing of mushrooms with 3D printed layer of cyanobacteria and graphene nanoribbons for the production of bioelectricity. Reprinted with permission from [12]. Copyright (2018) American Chemical Society.
